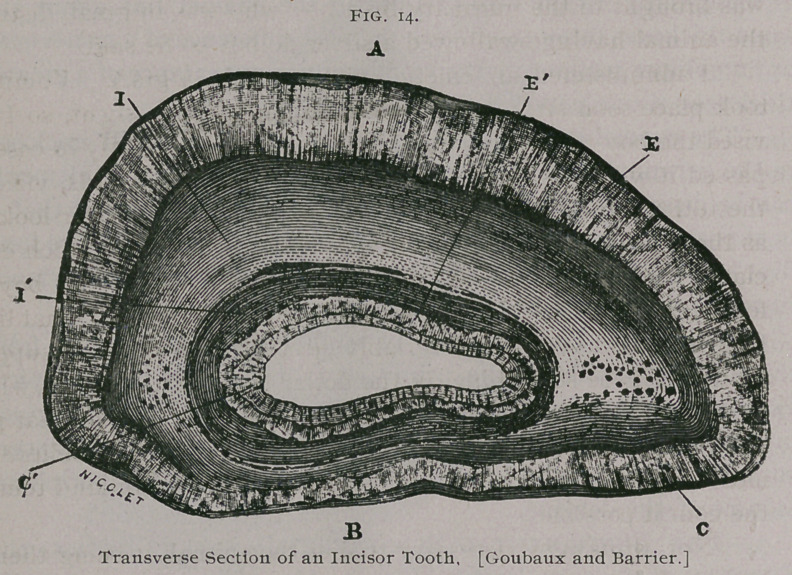# Age of the Horse, Ox, Dog, and Other Domestic Animals

**Published:** 1890-03

**Authors:** R. S. Huidekoper

**Affiliations:** Veterinarian


### AGE OF THE HORSE, OX, DOG, AND OTHER DOMESTICATED ANIMALS.

By R. S. Huidekoper, M.D., Veterinarian.

[*Continued from page 112.*]

*c. Structure of the Incisors.*

The study of the structure of the incisors of permanent dentition furnishes valuable indications for determining the age of the horse. This study is an indispensable adjunct for the complete appreciation of that which has preceded it, and will allow us to understand better the peculiarities of form which we find on the table of the tooth in its changes from year to year.

If we take as a type an inferior incisor at the moment of its first appearance in the body of the jaw bone, we find that it commences as a germ, which consists of a sack (germ follicle), into the interior of which protrude two papillæ ; one superior, *a*, and one inferior, *b*, Fig. 11), which look toward each other.

The first, *the germ of the enamel*, is of a conical form, and is placed in what will become the dental cup.

The second, *the germ of the ivory*, depressed from in front to behind, fills the cavity which hollows out the root of the tooth.

The surface of the first is destined to the formation of the enamel of the tooth, which it completes by the time the tooth has appeared through the gum, when the papilla disappears.

The other, which forms the ivory or dentine, remains until an advanced period of the life of the animal, and furnishes the tooth with its vitality.

In the interspace between these two papillary systems is deposited the matter which constitutes the tooth (*d*, Fig, 11). This at first consists of a very thin conical plate hollowed out on the inside, and contains a deep depression on its free extremity. Later, the walls of the dental follicle (*c*, Fig. 11), are transformed into the alveolar periosteum. If we refer now from Fig. 11, where we find *a*, the papilla of the enamel, *b*, the papilla of the ivory, *d*, the plate consisting of the deposits of these two papillaæ, that is, the enamel and dentine juxtaposited, and *c*, the germ sack, which is to become the alveolar peristoeum, to Fig. 9,

(Page 110), which is a longitudinal antero-posterior section of an inferior permanent pincher tooth, fully developed, somewhat enlarged, we find E, the enamel of the cup, surrounding this cavity from which the papilla has entirely disappeared; P, the cavity of the ivory papilla very much diminished in size; I, the dentine occupying the major portion of the tooth, having developed on the inner surface of the thin plate which we found in Fig. 11. In Fig. 9, at T, which represents the table of the tooth, after the borders have been worn, we find a separation of the enamel of the cup and the ivory enamel (Fig. 9, E E) which we find continuous in Fig. 11, *dd*.

FIG. 11.

Schematic section of the dental follicle of an inferior incisor in the horse. [Goubaux and Barrier.]

a, Superior papilla—germ of the enamel.

b, Inferior papilla—dental pulp or germ of the ivory.

c, Wall of the follicle.

d, Plate of enamel and denture juxtaposited.

Three substances enter into the structure of the teeth—one fundamental, the dentine or the ivory, and two covering substances, which differ from each other very much, and are known as the cement and enamel.

1. THE CEMENT (Fig 9 CC).—The cement forms the most superficial layer, and is deposited directly upon the enamel over the whole surface of the tooth, and dips into the cup, which it fills more or less completely in different subjects. Sometimes it is excessively thin, 2 to 3 mm.; at other times it is from 10 to 15 or even 20 mm. in thickness. It is generally thicker in the lower teeth than in the superior. MM. Chauveau and Arloing, in France, and Mr. Mayhew, in England, were the first to call attention to the importance of this in judging age.

It will be readily seen that the depth of the dental cup depends greatly upon the amount of the cement which is deposited in it. It is exceedingly rare to find excess of depth, due to increased depth of the enamel, which condition will, however, be considered in the study of the abnormalities of the teeth.

The cement is a protecting layer which offers only a moderate resistance to the friction of food and other substances, and disappears at an early date from the periphery of the tooth, while it persists in the cup of the tooth so long as the latter remains on

FIG. 12.

the table, where it forms a whitish spot, surrounded by a band of enamel. (Fig. 10, A, B, and C.) The cement is shown by microscopical examination to be a bony formation, secreted by the alveolar periosteum (germ sack), and is not a transformed ivory or dentine as given by Simonds.

It is found in greatest quantity at the crown or free extremity of the tooth, but in very old horses, when the teeth have worn down to the roots and the dentine, no longer protected by the

FIG. 13.

Antero-posterior and Longitudinal sections of an incisor tooth of horses of different ages, showing the gradual diminution of the pulp cavity. Goubaux and Barrier.]

enamel, offers a diminished resistance to the friction to which they are exposed, the irritation of the dormant periosteum sets up a new secretion, and cement is thrown out often in very great quantities to strengthen weak roots. As the inferior incisors are the shortest, and the first to wear away, we more frequently find the large masses of cement in the lower jaw. We again find excessive deposits of cement around the roots of the teeth when they have been loosened or irritated in their cavities by the rough pressure of bits, or have been injured by any accidental cause. This occurs most frequently in vicious or playful horses, who have the habit of biting roughly at foreign bodies. We see in this a wise provision of nature to succor these important organs after accident, and from the effects of old age.

2. The Enamel (Fig. 9, EE. Fig. 11, outer plate of d.)—The enamel is the true protecting layer of the teeth. Underneath the cement it forms a sort of armor which covers the surface of the dentine and forms the walls of the cup. It does not reach the cavity of the pulp. It is thicker, and covers a greater extent on the anterior face of the tooth than it does on the posterior. (Fig. 9, EE.) This furnishes an important factor in determining the age of very old horses, when the tooth becomes either triangular or biangular. In the wall of the cup the enamel has about the same thickness throughout, although the difference of a transverse, or an oblique section, may give a deceptive appearance of a greater thickness at one point than at another.

The enamel has a wonderful hardness. While still enclosed in the germinal sack it is readily cut by a knife and its elements can be dissected into **Z**-shaped prisms. From the moment that it is exposed to the air it becomes so hard that it will strike fire from flint. As it is more resisting than the dentine it constantly stands in relief on the surface of the table of the tooth.

*Histology.*—The enamel is of epithelial origin, formed from the superior papilla of the primitive follicle. On microscopical examination it is found composed of an infinity of little hexagonal prisms, intimately joined and directed obliquely to the subjacent surface. The deepest layers lie immediately over the peripheral lacunæ of the dentine.

3. The Fundamental Substance, Dentine, Eburnated Substance or the Ivory (Fig. 9, II).—This constitutes the major part of the tooth. It is produced by the inferior papilla or pulp, has a deep depression in its free extremity for the dental cup, and is covered by the enamel. It forms the walls of the pulp cavity. In the forming tooth the dentine consists of a thin layer juxtaposited to the layer of enamel formed by the superior papilla (Fig. 11, *d*), but as the tooth develops, successive layers are deposited on the interior of the first and the ivory gradually encroaches upon and diminishes the size of the papilla lodged in the pulp cavity until the latter entirely disappears.

The deeper layers have a darker color than the first, as in their continual infringement on the vascular papilla they imprison a portion of its organic matter and blood supply until they have produced a complete atrophy of their progenitor.

This discoloration of the dentine we will find later on the table of the tooth under the name of the "dental star," which becomes an important factor in judging of age after eight years. Strictly speaking, there are two stars, but the posterior is very rudimentary and of no practical importance. It is occasionally, however, seen very distinctly.

In Fig. 13 we have a series of longitudinal antero-posterior sections of an incisor tooth from horses of different ages, which show the gradual diminution of the pulp cavity from the encroachment of the dentine. The dentine towards the root of the tooth

FIG. 14.

Transverse Section of an Incisor Tooth. [Goubaux and Barrier.]

is always of a darker color than that towards the crown. The ivory is less hard than the cement, but much more resisting than either the cement or the bone in which the tooth is imbedded.

*Histology.*—The dentine is channeled by a multitude of *canaliculi* which radiate from the pulp and after frequent anastomoses terminate in lacunæ under the deep layer of the enamel (*interlobuar spaces of Czermak.*)

[TO BE CONTINUED.]

## Figures and Tables

**Fig. 11. f1:**
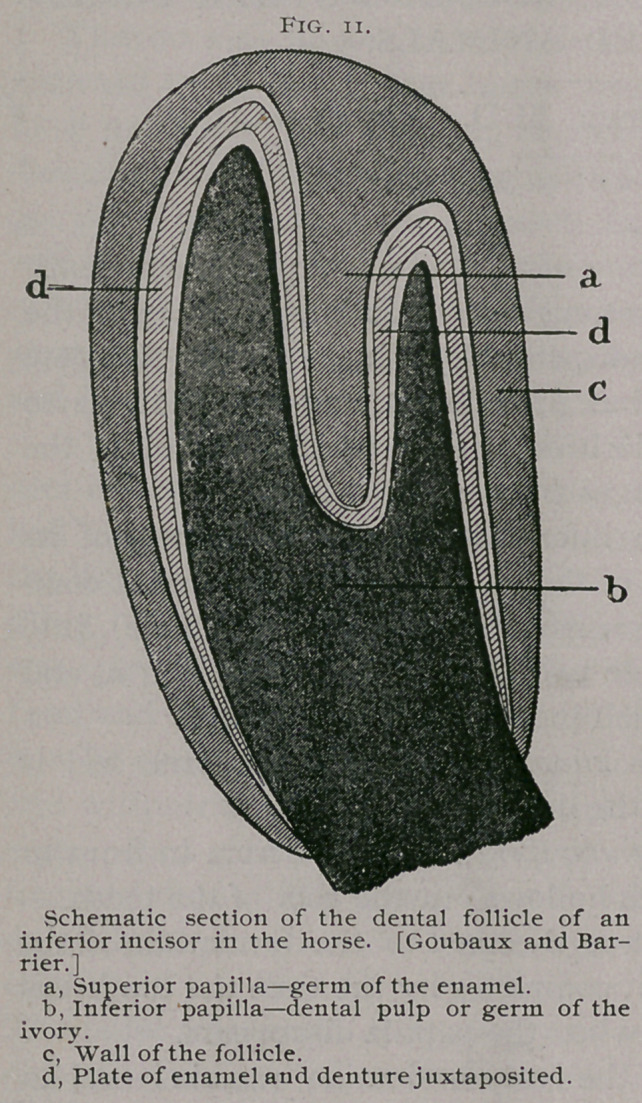


**Fig. 12. f2:**
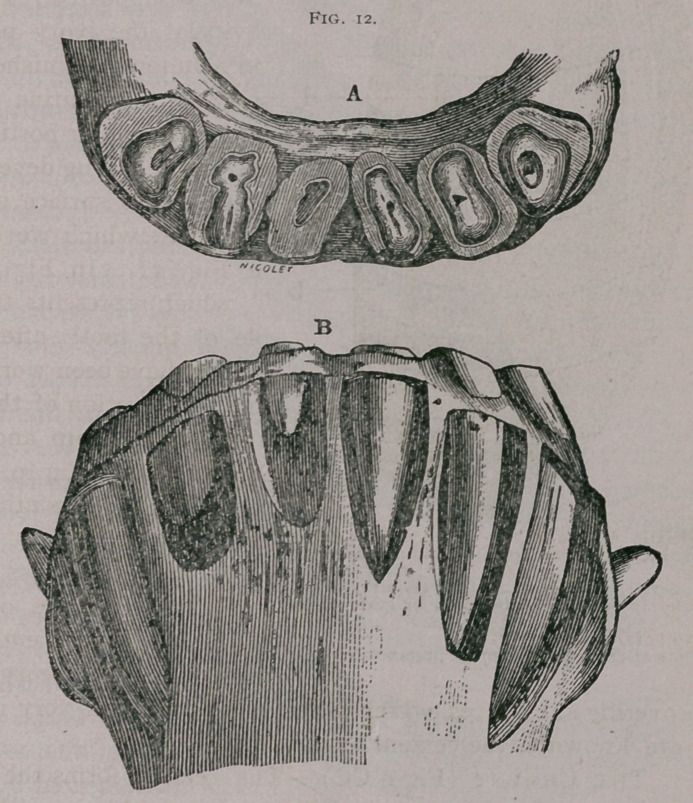


**Fig. 13. f3:**
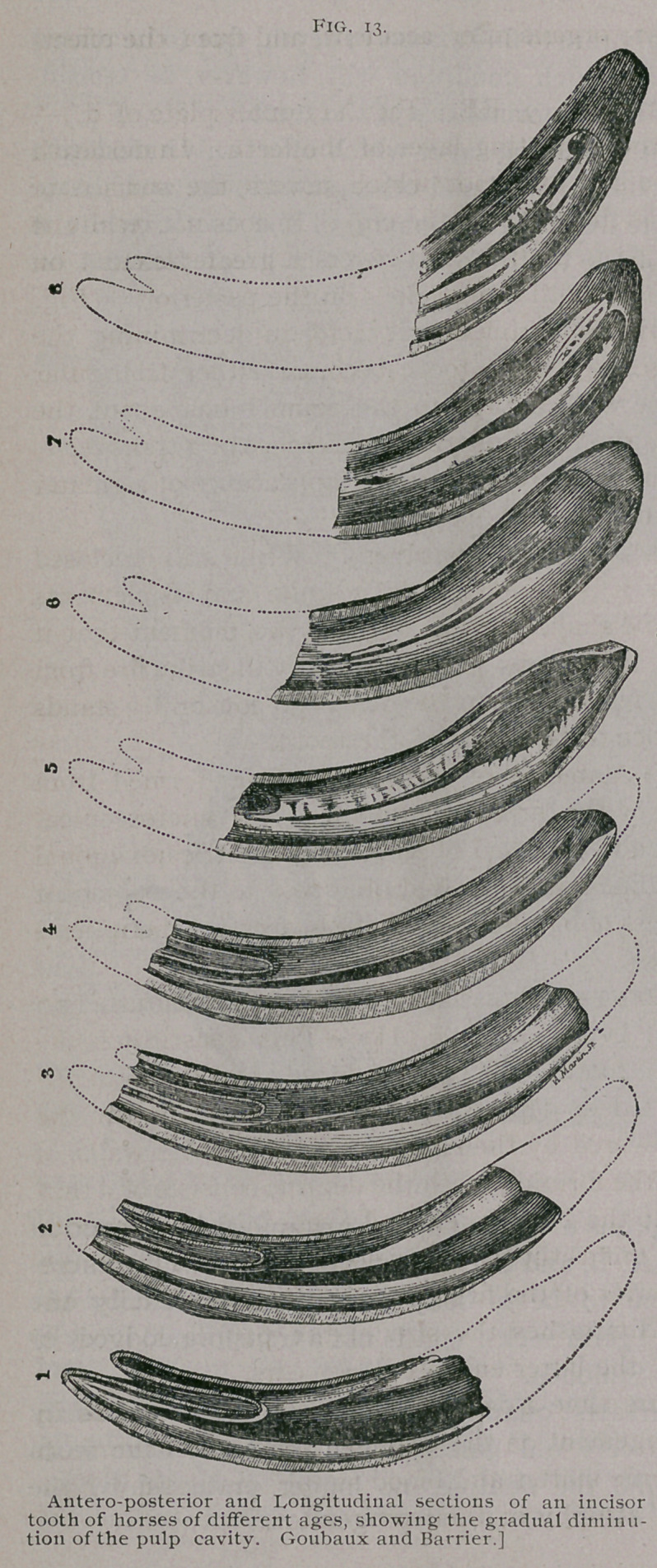


**Fig. 14. f4:**